# Evidence for functional state transitions in intensively-managed soil ecosystems

**DOI:** 10.1038/s41598-018-29925-2

**Published:** 2018-08-01

**Authors:** L. C. Todman, F. C. Fraser, R. Corstanje, J. A. Harris, M. Pawlett, K. Ritz, A. P. Whitmore

**Affiliations:** 1 Rothamsted Research, Harpenden, AL5 2JQ UK; 2 Cranfield University, Cranfield, Bedford MK43 0AL UK; 3 The University of Nottingham, Sutton Bonington Campus, Leicestershire, LE12 5RD UK

## Abstract

Soils are fundamental to terrestrial ecosystem functioning and food security, thus their resilience to disturbances is critical. Furthermore, they provide effective models of complex natural systems to explore resilience concepts over experimentally-tractable short timescales. We studied soils derived from experimental plots with different land-use histories of long-term grass, arable and fallow to determine whether regimes of extreme drying and re-wetting would tip the systems into alternative stable states, contingent on their historical management. Prior to disturbance, grass and arable soils produced similar respiration responses when processing an introduced complex carbon substrate. A distinct respiration response from fallow soil here indicated a different prior functional state. Initial dry:wet disturbances reduced the respiration in all soils, suggesting that the microbial community was perturbed such that its function was impaired. After 12 drying and rewetting cycles, despite the extreme disturbance regime, soil from the grass plots, and those that had recently been grass, adapted and returned to their prior functional state. Arable soils were less resilient and shifted towards a functional state more similar to that of the fallow soil. Hence repeated stresses can apparently induce persistent shifts in functional states in soils, which are influenced by management history.

## Introduction

Climate change is predicted to affect not only average climatic conditions but also their inherent variability^1^, exposing ecosystems to new disturbance regimes. Such changes have the potential to exceed resilience thresholds, causing ecosystems to transition to alternative states^2,3^. The complexity of natural systems, however, makes it challenging to predict when such transitions might occur and what the alternative functional states these systems might tend towards. In this study, we used soil microcosm experiments to investigate alternative stable states in complex ecosystems. The intention was twofold: (i) as soil functioning is critical to wider ecosystems and food production, to use these experiments to improve understanding of resilience in soils, and (ii) as microcosm experiments allow complex ecosystems to be exposed to different disturbance regimes, to use the experiments to develop a new approach to considering resilience in complex ecosystems more widely. To ensure differences between the soil ecosystems used in the microcosm experiments, we sampled soils from plots of in a long-term field experiment which have been managed under known land use (grass, arable and fallow) for over 50 years and the different land use treatments are known to have affected both the composition and functioning of the soil microbial communities^4,5^. To simulate the effect of a change in disturbance regime, the soil microcosms were exposed to a series of extreme drying and rewetting disturbances.

The concept of resilience thresholds builds on the idea that natural systems have evolved self-organising mechanisms that attract the system back towards an equilibrium state after a disturbance (e.g.^6,7^). Such equilibrium states could even involve a dynamic form in which cyclical behaviour occurs due to temporal cycles or periodic interactions within the ecosystem^8^. A resilience threshold thus represents the magnitude of disturbance that prevents self-organising mechanisms from acting so that, rather than returning to the prior equilibrium state after disturbance, the system tends instead towards an alternative equilibrium state. In some systems, alternative equilibrium states can be clearly identified based on an understanding of the system dynamics. The classic example is that of lake eutrophication^9,10^, for which two states are identified: either the water is clear, or it is turbid as a result of the eutrophication that has occurred. However, due to the dynamic and complex nature of most ecosystems, such distinct equilibrium states cannot be identified easily. Nevertheless, it is still likely that ecosystems have evolved self-organising mechanisms that act to respond to the variability of the external environment, or they would not persist. Yet there are also likely to be limits beyond which these self-organising mechanisms are less effective or cannot occur.

**Figure 1:**
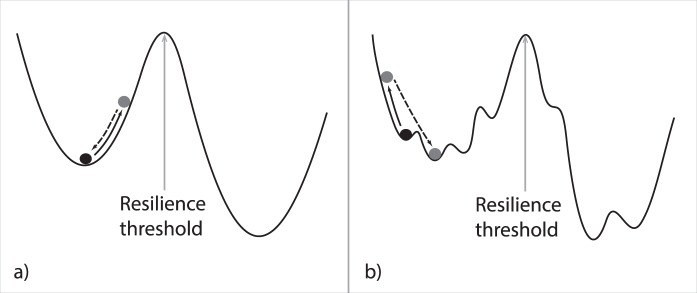
The concept of ecological resilience illustrated by considering a ball in a basin of attraction in (**a**) a simple model system and (**b**) a complex system with multiple stable states. Whilst the system exists in one particular state at a given time (the black ball) this state is dynamic and will change depending on the variability of the surrounding environment (the arrows and grey balls indicate how the state may change). Due to self-organising mechanisms the system state tends to stay within a range (the cup) unless a disturbance is sufficient such that a resilience threshold is exceeded, when the system instead tends towards an alternative stable state.

The concept of resilience thresholds is often illustrated using the analogy of a ball inside a cup (Fig. 1a). Whilst the ball represents the instantaneous state of the system, which changes continuously within dynamic ecosystems, the cup represents a ‘domain of attraction’ and the bottom of the cup represents the state towards which the system inherently tends^8^. Thus, after a disturbance that moves the ball, the shape of the cup relates to the expected response of the system, to be ‘attracted’ back down into the cup. However, if the ball is disturbed by a sufficiently extreme event, it may escape the cup and then move into an alternative domain of attraction^11^. The resilience may then be determined in terms of the magnitude of an event that causes the ball to ‘tip’ into the other cup. In a complex system, however, the stability landscape is also likely to look more complex (Fig. 1b) with multiple possible states, between which the system may move in response to environmental conditions. The system may move between some of these states easily in response to the characteristic dynamics of the surrounding environment. Other changes, however, could represent a change of state that is more difficult to reverse and that a resilience threshold has been exceeded. In complex systems, it is difficult to investigate the stability landscape and to quantify resilience thresholds because it is challenging to identify stable states. New methods are therefore necessary to identify states that complex systems are drawn towards, be these equilibrium states or recurring states in a dynamic cycle, and associate these with the potential risk of critical transitions in the system.

Soil resilience to disturbance is a topic of particular interest because of the role that soils play in underpinning the functioning of the Earth system, notably including agricultural production and thus ensuring adequate food production for a growing global population. Soils are important for carbon (C) and nitrogen (N) cycling; they can sequester C, potentially mitigating greenhouse gas released from other human activities, but also emit greenhouse gases themselves, and deliver a myriad of other ecosystem goods and services^12,13^. Soil microbial communities are particularly complex and dynamic, making it challenging to identify resilience thresholds, and there is concern over how soil processes will respond to changes in climate. Specifically, expected changes in climate variability would expose soils to new disturbance regimes. Research efforts have thus aimed to quantify and compare the resilience of soils (e.g.^14,15^). These studies generally focus on changes in a prescribed soil function after a disturbance^16–19^, using experimental observations of how a function responds to disturbance alongside metrics to quantify whether a soil function is maintained or recovers after a disturbance, how long the return to the prior functional state might take, or how close to the prior function the soil is able to return^20^. Studies have shown, however, that soil community structure and functions rarely return to their prior condition after a disturbance^21,22^. This is in some senses unsurprising due to the complexity of soil microbial communities – indeed it is possible that in complex communities a number of smaller-scale domains of attraction interact with larger scale mechanisms (Fig. 1b). Soil microcosm experiments, in which a highly complex community can be isolated and exposed to controlled conditions, thus provide a useful model system to observe the stability landscape within a complex community.

The state of an ecosystem is often defined in terms of the populations of each of the various species that are present. In microbial communities, however, the notion of species is less easily applied^23,24^ especially with regards to bacteria and archaea, given that many lineages have relatively simple morphologies and do not engage in reciprocal genetic recombination. Rather, there is a genetic continuum among bacterial lineages that is the result of recombination (acquisition of DNA fragments from closely related lineages^25^) and lateral gene transfer (acquisition of DNA segments from distantly related organisms^26^). In this work, therefore, the state of the microbial community is instead observed in terms of the ability of the soil to function, which we refer to as the ‘functional state’ of the associated soil. Soils have multiple functions which could be used to determine the functional state, and here we prescribed and observed a fundamental component of this functional state, i.e. the ability to metabolise a chemically complex organic substrate.

Land use is a particular cause for concern regarding soil resilience, as several studies have suggested that arable soils are less resilient than grassland soils when exposed to various disturbances^14,18,27^. Nevertheless, it has also been shown that soil microbial communities in arable soils quickly start to recover if they are converted back to grass^5^. In this study, we took soils from plots on the Highfield land-use change experiment at Rothamsted Research, and exposed them to 12 sequential cycles of drying and rewetting. The purpose was to go beyond observing the response to a single stress, but to observe whether under repeated stresses the soil adapted in order to be able to respond to such events. Land uses were long term grass meadow, wheat and bare fallow as well as two reversion treatments which had been established as either grass or wheat in 1959 and maintained for 49 years until 2008 when selected plots were ploughed and their land use swapped^5^. The difference in land use provided a context in which we expected to observe differences in resilience, whilst the use of established experimental plots ensured that the soil history was otherwise similar, potentially providing an opportunity to observe different functional states in the microbial communities.

We hypothesised that soils and functions mediated by soil communities would change with disturbance but that prior land-use might influence the nature and extent of these changes. Here we prescribed the function to be respiratory response to added substrate, since it is fundamentally related to collective microbial activity and is a central parameter with respect to C cycling in soil systems. In our experiments, we therefore observed the soil respiration response after the addition of barley shoot powder both prior to drying and after 1, 2, 3, 4, 7 and 12 cycles of drying and rewetting stresses. Thus, although the addition of substrate could in itself be considered a disturbance, here it was used as a prescribed measure of the functional state in order to quantify the functional changes after a series of drying and rewetting stresses. Changes in the respiration responses after increasing stress cycles were then measured and compared to see if the soils recovered their functional state after repeated disturbances (i.e. to observe the resilience to a change in disturbance regime). This comparison was made by fitting a descriptive numerical model to each of the respiration response curves. The model parameters were then used to describe key characteristics of each response (e.g. the timing and magnitude of different pulses). A cluster analysis was subsequently used to identify groups of responses with similar shapes and quantify how the shape of the response changed with increasing dry-wet cycles.

## Results

**Figure 2:**
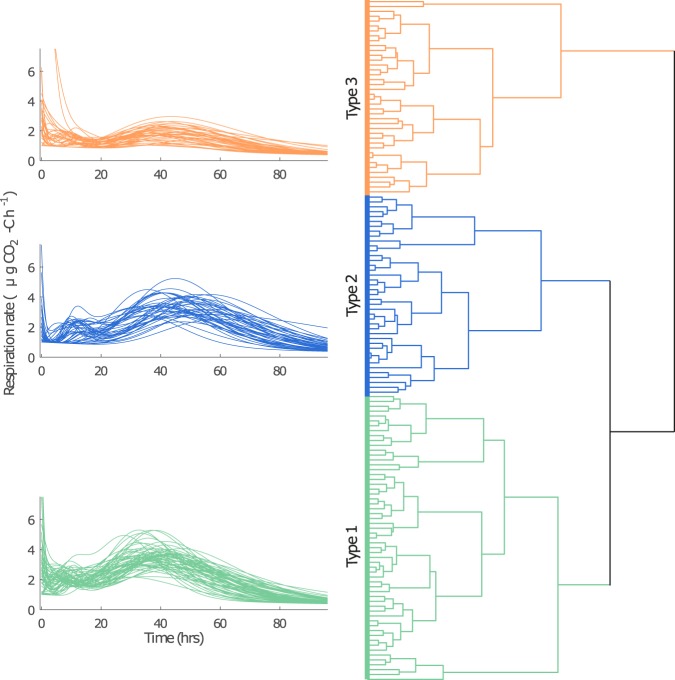
Hierarchical clustering of the set of model parameters identified by fitting the model to soil respiration in each replicate after each cycle. Three distinct types of response were identified; Type 1 with well defined initial decay and secondary and tertiary respiration pulses, Type 2 typically with smaller initial decay and a later tertiary pulse and Type 3 characterised by small secondary and tertiary pulses. On the left the fitted models for all of the responses of each type are shown.

**Figure 3:**
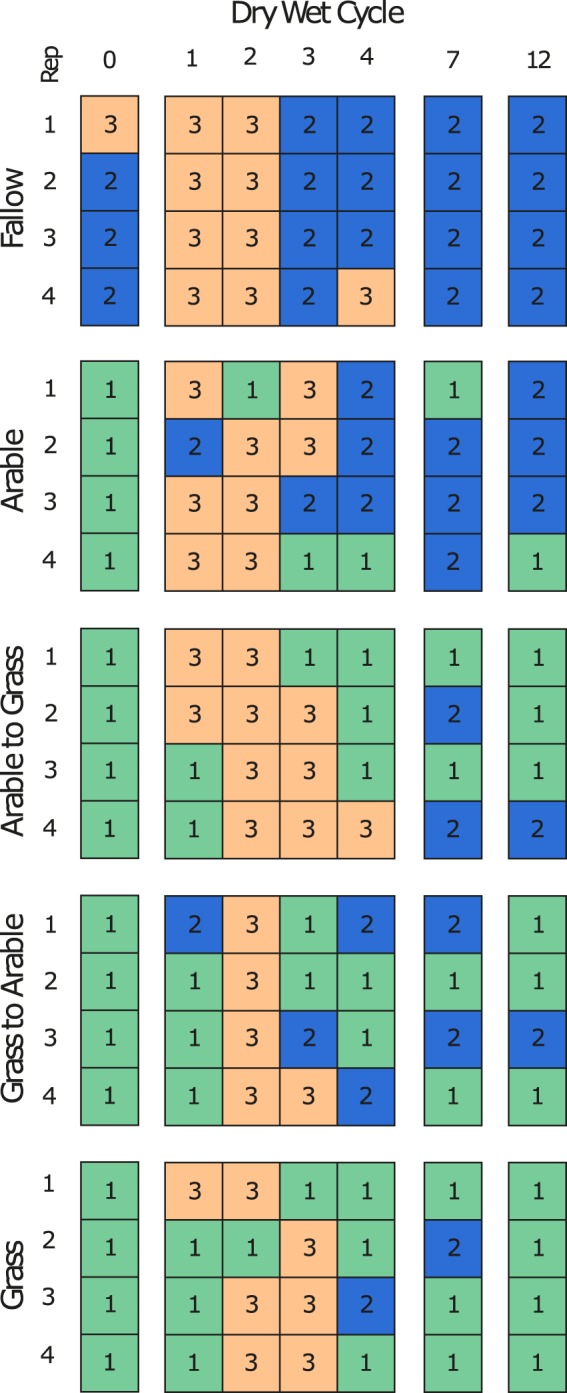
The types of respiration responses observed in response to substrate addition in different soil treatments, after an incubation period (cycle 0) and increasing cycles of drying and rewetting. The response type numbers and colour coding correspond to those identified by the hierarchical cluster analysis shown in Fig. 2. Cycle 0 shows the type of response prior to disturbance.

Soil respiration profiles in response to substrate addition were quantified using a modelling approach. This effectively summarised the shape of each profile curve in a series of parameter values thus allowing identification of differences between the soil respiration responses. A cluster analysis was used to compare all of the observed responses simultaneously, thus the analysis was based only on the shape of the respiration profiles not on the cycle or land-use from which the samples came. Soil respiration profiles in response to the addition of barley substrate clustered into 3 distinct types of response (Fig. 2), *viz*. Type 1, characterised by a high initial decay and well-defined secondary and tertiary pulses of respiration; Type 2, characterised by a smaller initial decay and a late tertiary pulse; Type 3, characterised by relatively small secondary and tertiary respiration pulses and thus a low total respiration. These characteristics were identified by comparing the model parameters of the different clusters (Fig. S1). Relating these types of responses to the replicate, cycle and land-use showed that multiple replicates frequently exhibited the same response type at the same time, revealing clear differences in the types of responses from soils originating from different land-uses as well as changes in responses after dry-wet disturbances (Fig. 3).

After the initial incubation period, prior to the first disturbance, the cluster analysis identified strong similarities in the response to the addition of barley powder in all the treatments except fallow (Fig. 3). All of the grass and arable soil responses behaved as Type 1. Thus, only the extreme land use (the fallow) appears to have resulted a change in the native soil community that is sufficient to produce a marked change in substrate mineralisation after the sampling disturbance and an incubation period.

All of the soils responded to the drying and rewetting disturbances via transitory Type 3 responses in which a reduction in respiration was observed (Fig. 3). This indicates that none of the soils were resistant to the change in disturbance regime we imposed, and that the resultant stresses were sufficient for us to observe the resilience of the soil function in terms of its ability to adapt after multiple stress cycles. For the soils from the grass plots, some replicates showed a transitory Type 3 response after the second drying and rewetting stress, rather than after the first stress, as in the arable and fallow plots. This was also true for the reversion plots (i.e. the grass plots that were previously arable and the arable plots that were previously grass). This indicates that the soils from the grass and reversion plots were more resistant to change induced by the disturbance regime.

After the transitory period, all of the soils’ responses returned to Type 1 or Type 2 responses that are characterised by more respiration than Type 3 (Fig. 3). This occurs despite the fact that the respiration responses are observed directly after disturbance within a dynamic regime, and repeatedly over multiple stress cycles suggesting that the soils had either reached a steady state or a steady dynamic state in which the cyclical response to dry-wet disturbances was steady. The return of the soils to Type 1 and 2 responses suggests that all of the soils had adapted to the disturbance regime in such a way that they were able to resist dry-wet disturbances and continue to process the carbon substrate. Notably, however, whilst most of the soil samples recovered to give similar responses to those observed prior to disturbance, the response in the arable soil after 4 cycles was more similar to that in the fallow soil (Type 2), and the arable soil remained in this functional state. A Type 2 response occurred in some replicates of the reversion plots (arable to grass and grass to arable) after more than 4 cycles. However in general, the soils currently under grass or the arable soil that had been under grass until 2008 were more able to recover their initial Type 1 response, and did so more quickly than the other land-uses. These results suggest that whilst the long term arable soils were not resilient to the changes in disturbance regime (as the prior Type 1 response was not recovered after repeated disturbances), the grass soils and arable soils that had been under grass until 2008 were more resilient because they adapted to the change in disturbance regime.

Responses within two particular replicates were markedly different to the others. In these two samples, much more respiration occurred within the first day after substrate addition compared to all of the other samples, and therefore the magnitude of the initial decay (B in Eq. 1) was around double that in any of the other soils. Both of these responses occurred in Cycle 2, one in an arable soil sample and the other in an arable to grass sample (see points labelled 3b in Fig. S2, Supplementary Information) and there was no known connection between the samples. The magnitude of these responses affected the cluster analysis, as including them reduced the emphasis on the initial decay in differentiating between the other soils. To consider the sensitivity of the results, a number of different cluster analyses were performed (details in Supplementary Information). This highlighted that most of the conclusions were robust to inclusion or exclusion of these outliers; specifically, the transitory cluster (Type 3 in Fig. 2) was identified in all clustering approaches, the ability of the grass soil to recover its prior state was identified in most analyses, as was a transition in the arable soil to an alternative state. However, the final state of the responses in the arable soil were not commonly clustered with those observed in the fallow. This suggests that, as well as some similarities between the final state of the responses in the arable soils and the initial and final states in the fallow, there are also additional, distinct differences.

Indeed, the clustering approach distinguishes the most distinct differences between the observed responses, and the analysis shown here identified 3 clusters. However, visual differences can still be observed between subgroups within these clusters. In particular, the cluster analysis further divides Cluster 2 into two subgroups, one associated primarily with the initial state of the fallow and the end state of the arable soils, and the other associated exclusively with the end state of the fallow (Fig. S2, Supplementary Information). As such, the results suggest that after multiple dry-wet disturbances the respiration response of the arable soil (immediately after rewetting) tends towards a response more similar to the response of the fallow prior to disturbance (Type 2a, Fig. S2), whilst the fallow response tends towards what is potentially another alternative functional state (Type 2b, Fig. S2).

## Discussion

On one level the results presented here are straightforward and provide support for a common conclusion, that grassland soils are more resilient to changes in climatic conditions than arable soils^11,15,20^. The results also provide strong evidence that previous management as grassland leaves a legacy that enhances resilience to a change in dry:wet disturbance regime, and that reversion to grassland can restore this resilience in other soils where it is previously lacking. In addition to these conclusions, the approach also highlights some of the challenges in operationalising the concept of resilience. For example, the fallow soil recovers a response that is comparable to its initial response and thus would be classed as ‘resilient’ along with the grassland soil. This illustrates the point made by Standish *et al*.^28^ that a resilient response is not necessarily synonymous with a ‘good’ response. If a system is already in an undesirable state it may actually be preferable that it changes after a disturbance. Here, no specific attempt has been made to identify what a desirable response to the addition of the carbon substrate should be. Given the extreme nature of the fallow treatment, however, and that the fallow response was initially distinctly different from all the other land use treatments, it is reasonable to assume that the fallow response was less desirable. Additionally, the soil resilience could have been quantified by considering the change in the total respiration induced by the addition of barley powder before and after disturbance. By this measure, all of the soils would be considered resilient as, despite a reduction in induced respiration in early cycles, after 12 cycles of drying and rewetting the total induced respiration was either comparable to, or higher than, that before any disturbance. Given the extremity of the imposed dry-wet stresses, this suggests that all of the soil microbial communities were highly adaptable because they all recovered their initial ability to process the carbonaceous substrate. As such, it is important to assess the soil response to repeated stresses and not just to a single extreme event if a change in disturbance regime is anticipated.

In this work a component of the functional state was observed by measuring the CO_2_ emitted from a soil after the addition of barley shoot powder. The assumption was that changes in this CO_2_ emission after stress occurred due to changes in the soil microbial community which resulted in a change in the soil respiration. This assumption is reasonable because previous work in which the same barley shoot substrate was added to 68 soils from across England and Wales showed that the shape of these respiration profile depended on both the physical soil structure and the associated microbial community, i.e. the soil ‘architecture’^29^. It the study we report here, it is unlikely that the soil structure changed significantly over the course of the experiment thus should not have affected the respiration profile, suggesting that changes in the respiration profile were due to changes in the microbial community. Additionally, from previous experimental and theoretical work, fluctuating dynamics of this kind are not unexpected after substrate addition to soils due to oscillations in populations of bacteria^30,31^. As substrate addition occurred at the same time as rewetting, however, and it has been shown that the resulting pulse of respiration may not be coupled with microbial growth^32^ changes in behaviour of the microbial community may also play role in the observed changes in respiration.

Soils are commonly observed to emit a pulse of CO_2_ after drying and rewetting and the source of this pulse is much debated^33,34^. A difference between the respiration profile after incubation and drying and rewetting cycles might therefore be expected. Nevertheless, the respiration from soil samples without added substrate was measured after each cycle and showed that there was a pulse of CO_2_ (Fig. S3, Supplementary Information), but that this was small relative to that emitted in response to substrate addition. It is therefore reasonable to assume that changes in the shape of the respiration profile occurred primarily due to changes in the soil microbial community over the course of the drying and rewetting disturbance.

In this work, soil respiration profiles in response to substrate addition were used to probe the potential existence of alternative functional states within soil microbial communities. Previous work^29^ showed that these respiration profiles after an incubation period were highly repeatable in the case of sub-samples from a previously bulked sample. Here, the respiration profiles observed in field-plot replicates after incubation were also highly repeatable. Thus the similarity did not occur due to the bulking of samples. Additionally, in prior work^29^, the form of the respiration profiles across a range of soils from England and Wales was similar enough to warrant a common descriptive model fitted for all soil types. A similar form was observed for the soils from Highfield, thus the same model was used.

Prior to the drying and wetting disturbances, the cluster analysis identified the same type of response to substrate addition for the soils from all land uses except the fallow. This corresponds to a prior observation that the fallow soil processes substrate differently to soil from the grass and arable plots^4^. Indeed, the land use change on Highfield has been shown to have a notable effect on the genetic structure of the soil microbial community^4,5^, particularly in terms of the abundance of the communities. Additionally, carbon inputs to the fallow soil are small because repeated ploughing (four times per year) prevents establishment of carbon-fixing weed biomass and has diminished soil structure over the years and thus the protection of carbon in the soil. Meanwhile, despite differences in field community structure, the repeatability of the results and the similarity across all the land uses except fallow suggests that common self-organising mechanisms exist within the communities. Thus, during the incubation period, the communities probably self-organised in response to the disturbance during the soil preparation period and were brought to similar functional states. This functional state could thus be considered as an attractor, towards which the soil microbial community in all of the soils except the fallow were drawn. The fallow, meanwhile, was drawn towards an alternative state.

When the disturbance regime started, the drying and rewetting cycles were sufficiently severe to affect the soil microbial response in all of the soils. The responses became more variable between replicates of the same treatment when the number of disturbances increased from 1 to 4. However, following subsequent disturbances (Cycles 7 and 12), the responses of soils from the grass and reversion treatments tended back towards their initial behaviour prior to disturbance. Thus, mechanisms within the soil acted as an attractor back towards this state. Meanwhile, the arable soil tended towards behaviour that was more characteristic of the fallow. These responses were also repeated across the replicates and maintained across multiple cycles (potentially between Cycles 4 and 12, although only three of these cycles were observed). This suggests that the substrate-induced respiration function had tended towards, and reached an alternative functional state.

The cluster analysis draws out the most marked differences between the different observed responses, but it does not explicitly identify all possible functional states of the soil. Indeed, within each of the clusters there are subgroups of responses that are distinctly different to other subgroups, and these differences could be considered to represent further alternative functional states. Nevertheless, the approach does suggest that there is more than one possible functional state in the soils after disturbance (response Types 1 and 2) and that, in the case of the arable soil, a change in disturbance regime can alter the attractor such that a different functional state is approached.

The experimental and modelling approach followed here suggests the existence of alternative functional states within soil microbial communities and further investigation of transitions between these states could allow the resilience thresholds approach to be applied to soils. Further research into the changes in the genetic and phenotypic microbial community structure and investigation of how this corresponds to the functional states would also be valuable. Additionally, further experimental work to observe any further change if the disturbance regime were to cease could elucidate the ease or rapidity with which the soil community can recover its prior state and thus whether the system has actually passed a resilience threshold.

Overall, the high degree of agreement between replicates prior to disturbance and the similarity of these functional response of soils from all land-use histories except for fallow indicates the existence of alternative stable functional states in soils. After the onset of dry:wet disturbances, soils from all land-use histories went through a similar, transitory response. We take this as strong evidence that the microbial communities have also changed. The clustering itself suggests different attractors in the responses to the repeated stress and we conclude that a history of grassland confers more resilience to a soil than a history of arable and that the more time spent recently in grassland, the more resilient the soil. Future research should (i) confirm by observation the expected change in the microbial communities, (ii) the nature of the benefit of grass land-management and (iii) quantify the duration of the benefit for different durations of land-management. This approach, based on analysis of results from a complex, but isolated ecosystem that could be manipulated and observed, also provides a tool to better understand resilience in complex biological systems.

## Methods

### Experimental

Soil samples were taken from the Highfield reversion experiment at Rothamsted Research, Harpenden, UK (Batcombe series, Flinty silt loam over Clay with flints, 51°80′N, 00°36′W, pH_H2O_ 5.9) in January 2016. Further details on the soils on these plots are provided in the Supplementary Information (Table S1). The Highfield experiment was established in 1949 as part of the so-called ley-arable experiments. In 1949 Highfield had previously been grass. Some plots remained as grass and others were ploughed and a continuous wheat crop was established. In 1959 an adjacent piece of land was fallowed (bare-fallow) and plants excluded by ploughing four times per year. In October 2008, these plots were further divided and while some remained under the previous management (i.e. continuous wheat, bare-fallow or grass), others were converted to one of the other management options (‘reversion plots’)^5^. For our purposes, samples were taken from 4 replicate plots of the following 4 treatment types; (i) continuous grass, (ii) continuous wheat, (iii) plots that had been planted with wheat until 2008 and then reverted to grass, and (iv) plots that had been grass until 2008 and then planted with wheat, each sample was maintained separately to give 4 replicates of each treatment. In addition to the continuous and reversion plots, samples were taken from 2 plots which have been under bare-fallow management since 1959, 2 sets of samples were taken from opposite sides of each plot to give 4 bare-fallow samples.

Soil cores from each plot were sieved to pass a 2 mm mesh. To determine the water content a subsample was dried at 105 °C for 48 h. The water holding capacity (WHC) was determined using the saturation and drain method modified from Harding and Ross^35^; briefly, a 20 g sample of sieved soil was placed in a funnel, saturated with 40 mL of de-ionised water for 30 min prior to being allowed to drain for a further 30 min. The volume of water drained was combined with the pre-determined water content to calculate the effective WHC. This was then used to adjust 200 g of each soil to 45% of their respective WHC. Prior to the experiment, water adjusted soils were then pre-incubated at 25 °C for 7 days to avoid artefacts caused by the disturbance of sampling and sieving.

For each plot, seven subsamples (0.5 g) of soil were weighed out. One subsample was mixed with 5 mg of freeze-dried powdered green barley (*Hordeum vulgare* L.) shoots, hereafter denoted ‘substrate’, thus supplying 2.25 mg C g^−1^ dry matter equivalent to the soil. Immediately following mixing, the 5 day time-course of CO_2_ evolution at 6-minute intervals was determined independently for each sample (those with added substrate and those without) using an automated multi-channel conductimetric respirometer (RABIT, Don Whitley, Shipley, UK^36^. Samples that had not received substrate were then exposed to up to 12 drying and rewetting cycles, where each cycle consisted of 3 days drying (enclosed in a sealed chamber with silica gel desiccant) followed by rewetting to 45% WHC for a further 4 days. Substrate was added to a sample (and the CO_2_ evolution determined) from each sample aliquot after 1, 2, 3, 4, 7 and 12 such drying and rewetting cycles. Sufficient experimental samples were set up so that each assay could be made destructively, i.e. barley powder was only ever added once to any sample.

### Modelling

**Figure 4:**
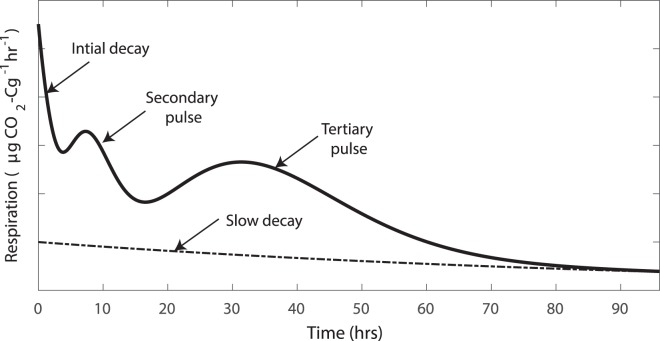
Illustration of the descriptive model of respiration that quantifies different features of the characteristic response via a series of parameter values.

A descriptive model was fitted to the respiration released from each of the soil samples after each substrate addition following the method described by Fraser *et al*.^29^. Briefly, this model describes the respiration (Y) in terms of different phases of the response, specifically an initial decay, followed by secondary and tertiary pulses of respiration, and a slower decay component (Fig. 4).

1 $$Y=B{e}^{-kt}+\frac{{A}_{2}{t}^{({\alpha }_{2}-1)}{e}^{-\frac{({\alpha }_{2}-1)t}{{\tau }_{2}}}}{{{\tau }_{2}}^{{\alpha }_{2}}{\rm{\Gamma }}({\alpha }_{2})}+\frac{{A}_{3}{t}^{({\alpha }_{3}-1)}{e}^{-\frac{({\alpha }_{3}-1)t}{{\tau }_{3}}}}{{{\tau }_{3}}^{{\alpha }_{3}}{\rm{\Gamma }}({\alpha }_{3})}+{e}^{-0.1t}$$

where *t* is the time after the start of the incubation, *B* and *k* are the magnitude and decay rate of the initial decay, *A*_2_ and *A*_3_ describe the magnitudes of the secondary and tertiary pulses respectively, *τ*_2_ and *τ*_3_ describe the time to the peak of the secondary and tertiary pulses, and *α*_2_ and *α*_3_ are shape factors that relate to the shape of the pulses. The parameters for the slow decay component are kept constant because differences between the respiration profiles from different soils are difficult to identify without a longer term observation of respiration.

The four terms in Y represent the following phases in the release of CO_2_ or decomposition of organic matter:

2 $$Y=Initial\,decay+Secondary\,pulse+Tertiary\,pulse+Slow\,decay$$

These phases observed in the responses might be associated with different groups within the soil microbial community or different components of the complex substrate that was added to the soil. Nevertheless, the model is not mechanistic and is instead used to formally characterise key features of the observed response profile, such as the time-to-peak and magnitude of the pulses of respiration observed after substrate addition (Fig. 4). This approach allows the shape of the curve to be summarised in terms of a set of 8 parameter values, with each parameter estimated for each replicate at each time that substrate was added to a soil sample by fitting the descriptive model to the observed respiration profile. Then, to improve the ease of interpreting what the shape factors describe, the parameters *α*_2_ and *α*_3_ were used to calculate the area under each of the pulses. These areas (the cumulative respiration during the secondary and tertiary pulses) alongside the model parameters (*B*, *k*, *A*_2_, *A*_3_, *τ*_2_ and *τ*_3_) thus described the shape of the respiration profile observed in each soil.

To compare the responses observed in different treatments and after drying and rewetting cycles, the fitted sets of parameter values were analysed using a minimum variance, hierarchical clustering approach following the Ward method^37^. All of the responses (from before disturbance and after subsequent cycles) were clustered simultaneously. This approach was used to group the response profiles (summarised in terms of the model parameter values) into those that were most similar to each other, also therefore identifying the largest differences between different response groups.

### Data availability

The datasets generated and/or analysed during the current study are available at, 10.6084/m9.figshare.5982337.

## Electronic supplementary material

Supplementary Information
