# Supplementary material for: Evidence for functional state transitions in intensively-managed soil ecosystems
Source: Sci Rep. 2018 Aug 1;8:11522. doi: 10.1038/s41598-018-29925-2 (PMC6070522; doi:10.1038/s41598-018-29925-2)
Supplement: Supplementary file 1 — Supplementary Information [file 41598_2018_29925_MOESM1_ESM.pdf]

Supplementary information for ‘Evidence for functional state transitions in intensively managed soil ecosystems’

Authors: LC Todman, FC Fraser, R Corstanje, JA Harris, M Pawlett, K Ritz, AP Whitmore

Soil Properties

Table S1: Soil properties (0-10 cm) of the Highfield experiment soil – a paleo-argillic brown earth, silty loam over clay (UK, Batcombe series), Chromic Luvisol (FAO) Latitude 51.80N, Longitude 0.36W. Typically the plots contain 150 g kg^{-1} sand (2000-63μm), 590 g kg^{-1} silt (63-2μm) & 260 g kg^{-1} Clay (<2μm)[a]

Land Use	Loss on ignition (%)[b]	Microbial biomass (C μg g^{-1} dw)[b]	Water holding capacity (ml g^{-1} dw)[b]	Organic Carbon (g C kg^{-1})[c]	Bulk Density (g cm^{-3})[d]
Fallow	1.8	71	0.28	9	1.30-1.45
Arable	2.4	179	0.29	16	1.30-1.45
Grass	5.4	532	0.32	45	0.99
Grass until 2008, then arable	3.9	300	0.38	28	n/a
Arable until 2008, then grass	4.3	462	0.33	22	n/a

[a]Measurements at the start of the experiment (Avery and Catt, 1995); [b] measurements from this study; [c] measurement for 0-10cm, 2012 (Hirsch et al., 2017).[d] measurements from 2008 (Gregory et al., 2016)

Cluster Characteristics

Typical cluster characteristics were identified by comparing the range of parameter values observed for the responses within that cluster.

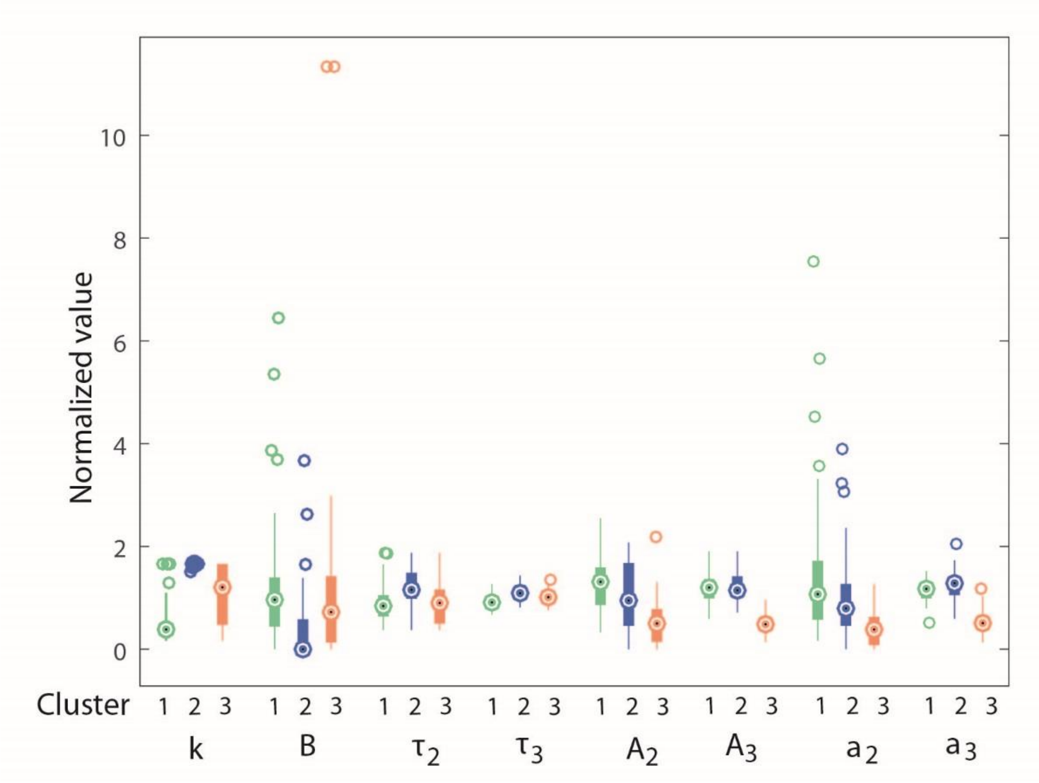

Figure S1: The typical value of parameters for the clusters, normalized relative to the mean of that parameter. The parameters B and k are, respectively, the magnitude and decay rate of the initial decay , τ_2 and τ_3 are the timing of the secondary and tertiary peaks, A_2 and A_3 are the amplitude of those peaks whilst a_2 and a_3 are the area under the secondary and tertiary peaks (which replace the shape factor parameters in the Eq 1 to give this parameter a more tangible interpretation). Means of the parameters used for normalisation were k = 0.60 h^{-1}, B = 1.76 µg CO_2-C h^{-1}, τ_2 = 10.7 h τ_3 = 42.3 h, A_2 = 0.86 µg CO_2-C h^{-1}, A_3 = 2.41 µg CO_2-C h^{-1}, a2 = 10.5 µg CO_2-C and a3 = 101.3 µg CO_2-C. The cluster numbers on the y-aixs correspond to those identified in the hierarchical clustering analysis in Fig. 3.

The Cluster Sensitivity

The structure of the model, in terms of the initial and slow decay along with the secondary and tertiary pulses, is imposed during the fitting process so that comparisons can be made across the range of responses made throughout the experiment. In certain responses, however, some of these components are less pronounced and the model struggled to identify parameter values. In these cases, the parameter values tended towards the bounds used to constrain the optimisation algorithm used for fitting. In particular, this occurred for many of the responses in Cluster 2, for which the initial decay was small. Thus the parameter for the initial decay rate was difficult to identify and tended towards the upper bound. The clustering approach, however, was still able to group these responses that were behaving in this similar way. In addition, two results in cluster 3 had a large magnitude of the initial decay (i.e. of parameter B in Eq. 1) and including them reduced the emphasis on the initial decay in differentiating between the other soils.

To test the sensitivity of the clustering results to the parameters for the initial decay the cluster analysis was carried out in a number of different ways. For example, (i) using the parameters for the secondary and tertiary pulses only (i.e. neglecting the parameters for the initial decay), (ii) neglecting two outliers with particularly large initial decay, which affected the standardisation of the decay parameters across the set (however, as these were considered to be due to the fungi that had been observed in these cases, there was a known reason for these outliers, so although they were different, they did form a part of the response set), (iii) fitting the results with smaller slow decay components (multiple values were considered). All of these differences had an effect on the cluster results, but there were common trends across the results that highlighted similar patterns to those observed in the analysis presented in the paper. Specifically, the results highlighted the similarity of the initial responses after the incubation period in all of the land uses except for the fallow and the similarity of the responses in the arable land type after 4, 7 and 12 cycles suggesting how stable this response state was. Some of the differences between the results of different clustering analyses are reconciled by observing the results using a different threshold to identify clusters, which identifies 6 clusters (Fig. S2). This corresponds to a sub-division of each of the clusters in Fig. 3 into 2 sub-clusters. These sub-clusters highlight that, after several dry wet cycles, the response of the arable soils to substrate addition tends to that of the fallow prior to disturbance, whilst the response of the fallow soil changes further.

The choice of model structure used in this paper was considered justified as it was identical to that imposed to analyse the responses of 68 soils from across England and Wales (Fraser et al. 2016a). In that experiment, despite the wide range of soils, the imposed structure did not struggle to fit the parameter for the initial decay rate. It was therefore interesting that it was challenging to identify this parameter in the responses in this study, particularly as some of the Highfield soils might be expected to be in poor condition such as an extreme land use (continuous fallow) and arable soils after repeated stress.

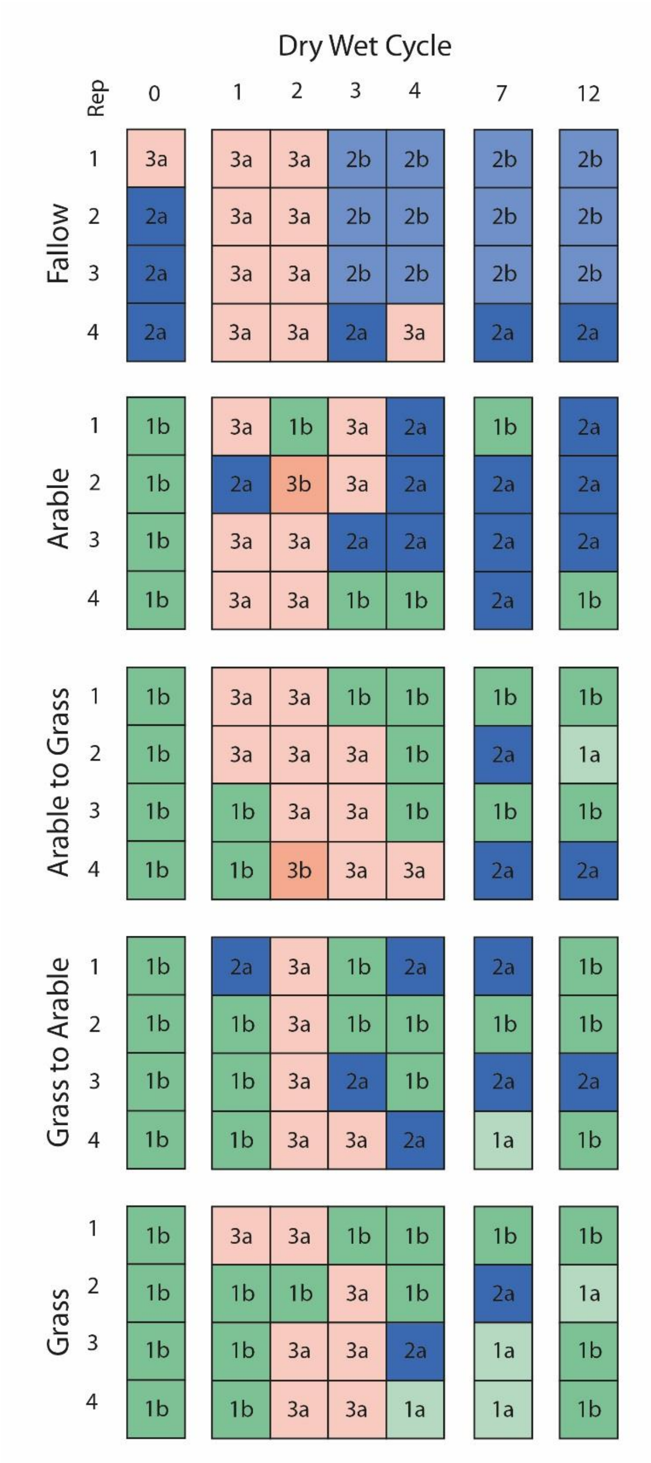

Figure S2: Results of hierarchical clustering of model parameter sets (that describe the shape of soil respiration profiles) observed with 3 cluster divided into 6 sub-clusters.

Birch Effect

An initial pulse of respiration is commonly observed after a soil is dried and rewetted. This is known as the Birch Effect (Birch, 1960). To quantify this effect, the CO_2 emitted from samples without added substrate was observed at the same time as respiration from those with added substrate. In this study the Birch effect was typically small (e.g. Fig. S3), occurring in only the first hour of the experiment. Occasional increases also occurred in some samples after several days, but were generally negligible in magnitude compared to the respiration observed from the samples with added substrate.

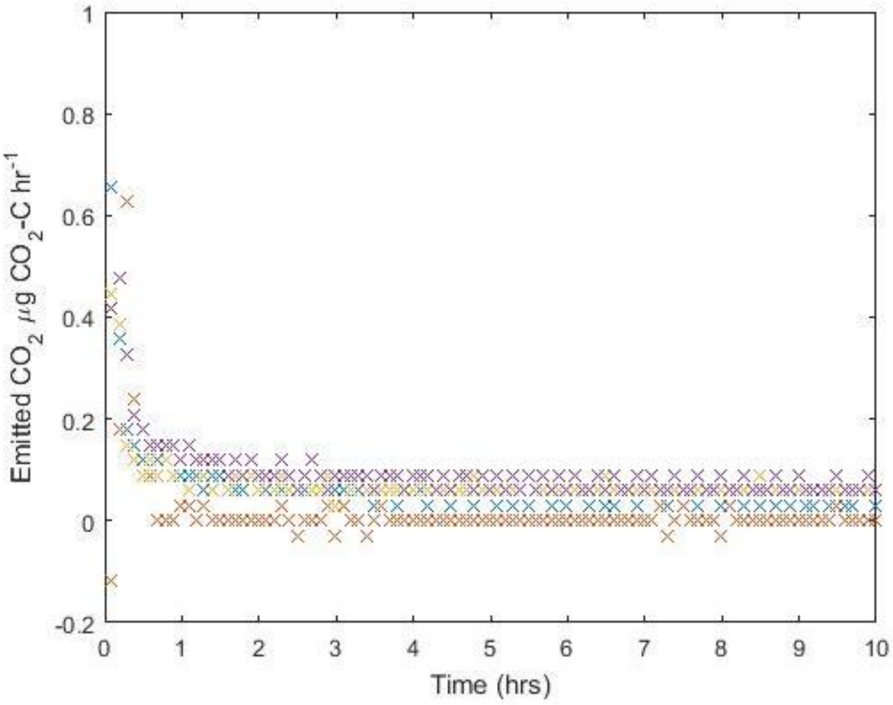

Figure S3: Example of Birch Effect (pulse of respiration observed due to drying and rewetting) observed for 4 replicates (red, yellow, blue and purple crosses indicate each replicate respectively) of the grass soil after the second drying and rewetting cycle.

References

Avery, B.W. & Catt, J.A. *The Soil at Rothamsted,* Lawes Agricultural Trust, Harpenden, UK. (1995).

Birch, H. F. Nitrification in soils after different periods of dryness. Plant and Soil **12**, 81-96. (doi:10.1007/BF01377763) (1960).

Fraser, F. C., et al. Distinct respiratory responses of soils to complex organic substrate are governed predominantly by soil architecture and its microbial community. Soil Biol. Biochem. **103**, 493-501. (doi: 10.1016/j.soilbio.2016.09.015) (2016).

Gregory, A. S., *et al.* Long-term management changes topsoil and subsoil organic carbon and nitrogen dynamics in a temperate agricultural system. European journal of soil science **67**, 421-430. (doi: 10.1111/ejss.12359) (2016).

Hirsch, P. R., *et al.* Soil resilience and recovery: rapid community responses to management changes. Plant Soil, 1-15. (doi: 10.1007/s11104-016-3068-x) (2017).
